# Workshop on Research Priorities for Management and Treatment of Angiostrongyliasis^1^

**DOI:** 10.3201/eid1812.120499

**Published:** 2012-12

**Authors:** Robert H. Cowie, James R. Hollyer, Alexandre J. da Silva, Robert G. Hollingsworth, Marlena C. Dixon, Praphathip Eamsobhana, LeAnne M. Fox, William L. Gosnell, Kathleen Howe, Stuart Johnson, Jaynee R. Kim, Kenton J. Kramer, Phaik Eem Lim, John F. Lindo, Zhao-Rong Lun, Arnaldo Maldonado, Alessandra L. Morassutti, Gerald S. Murphy, Sarah Y. Park, Yvonne Qvarnstrom, Ralph D. Robinson, Kittisak Sawanyawisuth, John Teem, Silvana C. Thiengo, Cheridah D. Todd, Hung-Chin Tsai, Gordon D. Wallace, Cecelia A. Waugh, A. Christian Whelen, Patricia P. Wilkins, Ting-Bao Yang, Hoi-Sen Yong

**Affiliations:** University of Hawaii at Manoa, Honolulu, Hawaii, USA (R.H. Cowie, J.R. Hollyer, J.R. Kim);; Centers for Disease Control and Prevention, Atlanta, Georgia, USA (A.J. da Silva, Y. Qvarnstrom, L.M. Fox., P.P. Wilkins),; Hawaii Department of Health, Honolulu (M.C. Dixon, S.Y. Park, A.C. Whelen);; John A. Burns School of Medicine, Honolulu (W.L. Gosnell, K.J. Kramer, G.D. Wallace);; Mahidol University, Bangkok, Thailand (P. Eamsobhana);; US Department of Agriculture, Hilo, Hawaii, USA (R. G. Hollingsworth);; Hilo, Hawaii, USA (K. Howe);; Loyola University Medical Center and Hines VA Hospital, Hines, Illinois, USA (S. Johnson);; University of Malaya, Kuala Lumpur, Malaysia (P.E. Lim, H.-S. Yong);; Sun Yat-Sen University, Guangzhou, People’s Republic of China (Z.-R. Lun, T.-B. Yang);; Instituto Oswaldo Cruz/Fiocruz, Rio de Janeiro, Brazil (A. Maldonado, Jr, S.C. Thiengo);; Pontifícia Universidade Católica do Rio Grande do Sul, Porto Alegre, Brazil (A.L. Morassutti);; Tripler Army Medical Center, Honolulu (G.S. Murphy);; University of the West Indies, Mona, Kingston, Jamaica (J.F. Lindo, R.D. Robinson, C.D. Todd, C. A. Waugh);; Khon Kaen University, Khon Kaen, Thailand (K. Sawanyawisuth);; Florida Department of Agriculture and Consumer Services, Tallahassee, Florida, USA (J. Teem);; and Kaohsiung Veterans General Hospital, Kaosiung City, and National Yang-Ming University, Taipei, Taiwan (H.-C. Tsai).

**Keywords:** Angiostrongyliasis, rat lungworms, parasitology, rats, snails, slugs, prevention, epidemiology, Angiostrongylus cantonensis, nematodes, Hawaii, USA, parasites, treatment, workshop

An international transdisciplinary workshop on angiostrongyliasis was held August 16–18, 2011, in Honolulu, Hawaii, USA, expanding on an inaugural workshop in Thailand in 2010 (*1*,*2*). The workshop convened scientists and clinicians from regions and countries as far apart as Brazil, China, Jamaica, Taiwan, Thailand, and the United States, with expertise in a range of fields including parasitology, ecology, food safety, epidemiology, diagnosis, and treatment. The workshop’s goal was to develop a rigorous research agenda to address the parasitic infection known as rat lungworm disease at a global scale through advancing an integrated understanding of the infection.

Angiostrongyliasis, which is caused by ingestion of uncooked/undercooked food containing third-stage larvae of *Angiostrongylus cantonensis*, the rat lungworm (Nematoda), is associated with eosinophilic meningitis (*3*). Common sources are infected snails and slugs as well as infected paratenic hosts (e.g., freshwater shrimp, certain fish). Snails and slugs (intermediate hosts) become infected by ingesting first-stage larvae from feces of rats (definitive hosts). These larvae then develop to third-stage larvae in the snails/slugs. The natural cycle continues when rats eat these hosts. The third-stage larvae migrate to the central nervous system, specifically the brain, where they mature to the fifth stage (young adults). They then return to the circulatory system where they reproduce in the pulmonary artery. Eggs are carried to the lungs, hatch, and move up the trachea and down the gut to be released in the feces.

In humans, after the larvae are ingested, the cycle breaks when the worms reach the fifth stage, typically in the brain, where they die (*3*). The physical damage caused by worms moving within the brain and the intense inflammatory immune reaction stimulated, especially by dead or dying worms, are thought to be responsible for the symptoms of angiostrongyliasis, which can lead to coma and, rarely, death.

Angiostrongyliasis is mainly a tropical disease, but with the increasing spread of invasive species, including rats and mollusks (*4*), and global warming, which may increase the hosts’ potential range, it has become an important emerging infectious disease. The link between human infection by this nematode and manifestation of eosinophilic meningitis was first recognized in Hawaii in 1961 (*5*); the stimulus for this workshop was an increase in recent cases (*6*). The keynote address, by a key researcher of that time (*7*,*8*), summarized the history of angiostrongyliasis in Hawaii. The workshop then continued with presentations that dealt with the biology, life cycle, and hosts of *A. cantonensis* lungworms. Key points made by the speakers were that many taxonomically unrelated snail/slug species, including temperate species introduced into tropical regions, can act as intermediate hosts and that a wide range of mammals, birds, fish, and invertebrates may be infected as accidental hosts or serve as paratenic hosts.

The second session summarized the global status of angiostrongyliasis, with presentations by participants from China, Taiwan, Thailand, Jamaica, Brazil, and the United States, including Hawaii. The disease is particularly widespread in China and Thailand, especially where raw snails, notably, introduced apple snails, are considered a delicacy (*1*,*9*,*10*). The potential role of slime-contaminated vegetables in transmission in Jamaica was also suggested.

The next sessions focused on diagnostic and clinical aspects. Presentations included clinical descriptions of the disease and clinical reports of cases of angiostrongyliasis acquired in Jamaica and Hawaii. Issues regarding the role of the immune response in the pathophysiology of infection were described. Reports on the current state of immunodiagnosis and future expectations for diagnostic methods noted the identification of the 31-kDa protein as a glycoprotein complex (the main antigen for angiostrongyliasis diagnosis). Presentations on detection of *A. cantonensis* lungworms in the environment described dramatically increased incidence in China and Thailand and increased cases in the Caribbean, Pacific Islands, Europe, and North America (*9*), methods for sample collection in hosts, and detection methods, including molecular (PCR) assays, which triggered discussion of the potential diagnostic use of PCR.

The next focus was on relative risks of infection and host control methods. The primary pathway of infection is the ingestion of raw snails or slugs, either deliberately or accidentally on contaminated food (*10*). Laboratory studies show that snail/slug slime may contain infective larvae, but no studies have confirmed this mode of infection, and the risk for infection is probably low because worm density is much lower than in the mollusks themselves. Infection by drinking contaminated water (caused by an infected snail or slug drowning in it) or through broken skin has not been documented. Presentations on disease control efforts in China and Hawaii emphasized control of snails and slugs rather than rats.

An acute need exists to raise awareness of angiostrongyliasis within the medical community and by the public. Opportunities for information dissemination include the Internet, press releases, and traditionally published review articles. Highly visible, large-scale clinical trials, perhaps in China or Pacific Islands, were suggested. Diagnostic considerations and options for clinicians include a preliminary standardized protocol, involving clinical signs, case inclusion criteria, and lumbar puncture findings. A reliable, rapid test is needed, possibly consisting of performing PCR on cerebrospinal fluid. In Hawaii, factsheets have been distributed (*11*), but outreach efforts have faced difficulties because farmers (and retailers) do not want their produce associated with a potentially serious illness. A series of community forums has been initiated to distil the information from the proceedings to inform the wider public.

In a concluding session of the workshop, the participants developed a list of 115 research and outreach needs, outlining the top 5–7 needs in each of 8 areas (Table). For complete information, including presenter details and abstracts, visit the workshop website at www.hawaii.edu/cowielab/Angio%20website%20home.htm.

**Table:** Research and outreach needs for management and treatment of angiostrongyliasis

Detection of *Angiostrongylus* *cantonensis* rat lungworms in hosts
• Genomics/proteomics: sequence genome/develop proteomics for fast detection
• Obtain comparative data on sensitivity and specificity of available techniques
• Develop methods of parasite detection in fresh human food, mainly vegetables and fruits
• Sample other potential hosts, notably flatworms and freshwater crustaceans, to assess their potential as hosts and their parasite load
• Develop low-technology detection methods
• Gain a better understanding of the biology of the hosts as it relates to parasite transmission
Control of hosts in the field (rats, slugs/snails, paratenic hosts)
• Identify paratenic hosts, their relevance, and their importance
• Gain a better understanding of the basic biology of snails and slugs, including genetics, which could be useful in developing interventions
• Undertake surveys of rats in areas where *A. cantonensis* lungworms have been reported (e.g., south Florida, Rota)
• Develop cultural methods of snail/slug control, such as natural barriers (e.g., sand)
• Gain a better understanding of the environmental variables that affect slug and snail host survival and reproduction, e.g., humidity, temperature, etc., and the potential effects of climate change/global warming
Public education to minimize chance of infection
• Involve children (ages 7–14) in educational efforts and build education about angiostrongyliasis into science/math curriculum (in the United States, there may be a National Science Foundation GK-12 grant opportunity)
• Require continuing education for health care practitioners
• Better define risk factors so that these can be the focus of education
• Increase outreach to farmers and farmers’ markets. Focus on potential impact on profits
• Use social media networks, e.g., Facebook, Twitter, etc., and contribute regularly
• Define public health messages clearly and consistently
• Create an angiostrongyliasis listserve
Control of hosts/larvae on produce (e.g., washing/rinsing)
• Evaluate different rinse ingredients
• In the United States, obtain Environmental Protection Agency and/or Food and Drug Administration approval of methods for washing produce; similarly, in Hawaii, obtain approval from the Departments of Agriculture and Health, as well as other regulatory agencies
• Undertake surveys to ascertain the distribution of larvae and hosts, including slugs/snails on different kinds of fresh produce
• Develop a hand-held loop-mediated isothermal amplification device or other simple methods for detection of *A. cantonensis* lungworms in the field
• Investigate irradiation of produce as a sanitizing method
Diagnosis
• Improve and standardize serology
• Develop rapid tests for detection, e.g., PCR, antigen detection, chromatography, dipsticks
• Standardize clinical criteria for diagnosis
• Validate PCR or other molecular methods for detection of *A. cantonensis* lungworms in patients
• Develop a cooperative network for sharing specimens, antigens and DNA sequences
Treatment
• Undertake well-thought-out clinical trials
• Assess the value of early use of anthelmintics
• Standardize the protocol for lumbar puncture (e.g., are serial/repeat tests beneficial; how often should they be done?)
• Develop guidelines for the use of steroid therapy, e.g., when to start, dosage, rate of tapering off
• Determine standard of care
Pathophysiology
• Determine the actual mechanism of neurologic injury in humans: (i) increased intracranial pressure, (ii) the inflammatory reaction, and if so which cytokines are involved, (iii) mechanical damage from worm migration, or (iv) a combination of these
• Develop the best animal model for human disease
• Assess the influence of parasite inoculum on incubation period and severity of the illness, in particular how the number of parasites in the inoculum correlates with the number of parasites reaching the brain
• Determine how the parasites invade the central nervous system (CNS)
• Determine at which larval stage intervention (anthelmintics) will prevent symptoms
• Investigate pathophysiology in infected hosts (slugs/snails, rats, paratenic hosts)
• Investigate the mechanism by which steroid treatment alleviates symptoms: inflammation reduction or reduction of intracranial pressure
Epidemiology
• Refine understanding of risk factors
• Develop better tools for molecular epidemiology
• Standardize the methodology of environmental assessments in terms of location characteristics and the geographic distribution of the parasite and the disease in a region
• Develop centralized reporting of epidemiologic findings
• Determine the relationship between infection and disease: what triggers the disease, how the level of exposure is related to incidence of the disease
